# Early Endosomal Trafficking Component BEN2/VPS45 Plays a Crucial Role in Internal Tissues in Regulating Root Growth and Meristem Size in Arabidopsis

**DOI:** 10.3389/fpls.2020.01027

**Published:** 2020-07-09

**Authors:** Yuki Matsuura, Narumi Fukasawa, Kosuke Ogita, Michiko Sasabe, Tatsuo Kakimoto, Hirokazu Tanaka

**Affiliations:** ^1^Department of Biological Sciences, Graduate School of Science, Osaka University, Toyonaka, Japan; ^2^Department of Life Sciences, School of Agriculture, Meiji University, Kawasaki, Japan; ^3^Department of Biology, Faculty of Agriculture and Life Science, Hirosaki University, Hirosaki, Japan

**Keywords:** Arabidopsis, auxin, root meristem, Brefeldin A, PIN-FORMED1, *trans*-Golgi network, early endosome

## Abstract

Polar auxin transport is involved in multiple aspects of plant development, including root growth, lateral root branching, embryogenesis, and vasculature development. PIN-FORMED (PIN) auxin efflux proteins exhibit asymmetric distribution at the plasma membrane (PM) and collectively play pivotal roles in generating local auxin accumulation, which underlies various auxin-dependent developmental processes. In previous research, it has been revealed that endosomal trafficking components BEN1/BIG5 (ARF GEF) and BEN2/VPS45 (Sec1/Munc 18 protein) function in intracellular trafficking of PIN proteins in Arabidopsis. Mutations in both *BEN1* and *BEN2* resulted in defects in polar PIN localization, auxin response gradients, and in root architecture. In this study, we have attempted to gain insight into the developmental roles of these trafficking components. We showed that while genetic or pharmacological disturbances of auxin distribution reduced dividing cells in the root tips and resulted in reduced root growth, the same manipulations had only moderate impact on *ben1; ben2* double mutants. In addition, we established transgenic lines in which BEN2/VPS45 is expressed under control of tissue-specific promoters and demonstrated that BEN2/VPS45 regulates the intracellular traffic of PIN proteins in cell-autonomous manner, at least in stele and epidermal cells. Furthermore, BEN2/VPS45 rescued the root architecture defects when expressed in internal tissues of *ben1; ben2* double mutants. These results corroborate the roles of the endosomal trafficking component BEN2/VPS45 in regulation of auxin-dependent developmental processes, and suggest that BEN2/VPS45 is required for sustainable root growth, most likely through regulation of tip-ward auxin transport through the internal tissues of root.

## Introduction

Plant hormone auxin is involved in multiple aspects of plant growth and development, such as embryonic patterning, formation of lateral organs, regulation of organ growth, and tropic responses (Mockaitis and Estelle, 2008; Vanneste and Friml, 2009; Grunewald and Friml, 2010; Zwiewka et al., 2019). Auxin-dependent developmental regulation largely relies on local auxin gradient, which is generated by local auxin biosynthesis, polar transport, and/or degradation (Vanneste and Friml, 2009; Casanova-Sáez and Voß, 2019). Root system plays physiological roles such as assimilation and transport of nutrients, and therefore, its architecture is an agriculturally important trait. Root system architecture (RSA) is typically determined by the extent of root growth and branching patterns, which are plastically modulated by external stimuli. Transport and local accumulation of auxin play pivotal roles in regulating both root growth and branching patterns. While certain level of auxin is required for root growth, excess amounts of auxin strongly inhibit root elongation, and promote lateral root initiation and emergence (Petricka et al., 2012; Sugawara et al., 2015; Banda et al., 2019). Auxin is mainly synthesized in young leaves and root tip, and shoot-driven auxin is transported through vasculature toward root tip (Ljung et al., 2005). At the root tip, auxin flux is redirected shootwards through the outer layer of tissues such as epidermis, cortex, and lateral root cap. This auxin flow depends on auxin influx and efflux transporters. Several classes of auxin transport proteins, including PIN-formed (PIN) family protein and AUXIN/LIKE AUX1 (AUX/LAX) family protein, have been isolated. Of these, PIN family auxin-efflux proteins are expressed widely in plant tissues, and some are localized to the plasma membrane (PM) with polar distribution and play a critical role in intercellular transport of auxin. Polar localization of PIN proteins are consistent with the known directionality of auxin transport (Tanaka et al., 2006) and in some cases, manipulation of PIN polar localization causes changes in auxin distribution (Wiśniewska et al., 2006; Huang et al., 2010), indicating the biological significance of the polar localization of PIN proteins in regulating auxin distribution.

In *Arabidopsis*, PIN1, 2, 3, 4, and 7 are localized at the PM (Grunewald and Friml, 2010). PIN1 is expressed in internal tissues in the root tip, and localized at the basal side of the PM. On the other hand, PIN2 is expressed in outer tissues, including epidermis, cortex, and lateral root cap, and is typically localized to the apical side of the epidermal cells. PIN3, 4, and 7 are expressed in the vasculature and columella cells. Members of PIN proteins have auxin efflux activity and expressed in partially overlapping patterns (Vanneste and Friml, 2009; Sauer and Kleine-Vehn, 2019). Due to their partial redundancy, the single mutants display specific defects in shoot organ formation, gravitropic defects or moderate patterning defects, whereas the multiple mutants exhibit remarkable inhibition of root architecture (Friml et al., 2003; Blilou et al., 2005; Vieten et al., 2005). The developmental defects of the *pin* mutants correlate with auxin distribution defects, which are consistent with the polar localization of corresponding PIN proteins at the PM, suggesting that PIN-dependent auxin transport plays essential roles in multiple developmental processes.

The polar localization of the PIN proteins at the PM requires post-translational modification of PIN proteins and membrane trafficking (Zwiewka et al., 2019; Zhang et al., 2020). For instance, in the root vascular tissues, PIN1 proteins are reversibly accumulated in endosomes and depleted from the PM upon treatment, when seedlings are treated with the vesicle transport inhibitor brefeldin A (BFA) (Geldner et al., 2003; Zwiewka et al., 2019). This indicates that PIN1 is constitutively transported by endocytosis and recycled back to the PM. Many membrane trafficking factors related to this trafficking have been isolated. GNOM ARF GEF is a prominent target of BFA in terms of PIN1 recycling to the PM and is required for localization of PIN1 at the basal side of the vascular cells (Geldner et al., 2003; Kleine-Vehn et al., 2008). In addition, ARF GEF interacting proteins such as ARF1 and Aminophospholipid ATPase3 (ALA3) play essential roles in the localization of PIN proteins at the PM (Tanaka et al., 2014; Singh et al., 2018; Zhang et al., 2020). BFA treatment induces the formation of agglomerated membrane compartments, where most of GNOM proteins accumulate. It is believed that endocytosed vesicles rapidly reach the *trans*-Golgi network which is equivalent to early endosome in plants (TGN/EE). BFA compartments are characterized by accumulation of many TGN/EE-related markers and therefore endocytosed as well as newly synthesized PM cargo proteins accumulate there. As such, it is hypothesized that BFA inhibits the trafficking from the TGN/EE in Arabidopsis. However, in the normal condition, majority of GNOM proteins are detected at the Golgi apparatus and at the PM, but not from the TGN/EE (Naramoto et al., 2014) and the molecular mechanism underlying trafficking and polar localization of PIN proteins is not fully understood.

In previous study, we isolated Arabidopsis mutants designated as *bfa-visualized endocytosis defective1* (*ben1*) and *ben2* mutants, which exhibited less pronounced PIN1-GFP accumulation at the BFA compartment by a fluorescence imaging-based forward genetic screening (Tanaka et al., 2009). *BEN1* encodes ARF GEF BIG5 (Tanaka et al., 2009), which activates ARF GTPases promoting membrane budding (Singh et al., 2018). *BEN2* encodes a Sec1/Munc18 protein VPS45, which interacts with SNARE protein to promote membrane fusion (Tanaka et al., 2013). Both BEN1 and BEN2 proteins localized to the TGN/EE and are speculated to functions in recycling of PIN protein. Mutation in either BEN1 or BEN2 moderately affects trafficking and polar localization of PIN proteins, but does not cause severe developmental defects. On the other hand, *ben1; ben2* double mutant shows pleiotropic defects including short primary root, excess number of lateral roots, and small shoot. However, detailed molecular mechanism by which BEN1 and BEN2 regulate root architecture still remain to be elucidated.

In this study, we attempted to dissect the role of the endosomal component BEN2/VPS45 in regulating PIN trafficking and root development. We showed that the meristematic activity is reduced in *ben1; ben2* double mutant and the root growth of the double mutants were relatively insensitive to genetic or pharmacological manipulation of auxin transport and synthesis. Furthermore, tissue-specific rescue experiments demonstrated that, while BEN2/VPS45 regulates intracellular trafficking of PIN1 and PIN2 in cell autonomous fashion, it is mainly required in internal tissues to promote root growth.

## Materials and Methods

### Plant Materials and Growth Conditions

Mutants and transgenic marker lines used in this experiment have been described previously: *ben1-1*, *ben2* (Tanaka et al., 2009), *pVPS45::VPS45-GFP* (Tanaka et al., 2013), *CycB1;1-GUS* (Colón-Carmona et al., 1999). *DR5rev::3xVenus-N7* (Heisler et al., 2005) was crossed with Col-0 two times.

Seeds were sterilized in 70% ethanol and rinsed with 100% ethanol. Then they were germinated and grown on 0.4% phytagel-solidified half-concentration Murashige and Skoog (MS) medium supplemented with 1% sucrose (pH 5.9) at 22°C.

### Plasmid Construction and Transgenic Plants

To generate pGreen-pPIN1::BEN2-GFP and pGreen-pPHB::BEN2-GFP constructs, a 2.3-kb *PIN1* promoter and a 3.0-kb *PHB* promoter were PCR amplified, and cloned in a GUS-GFP vector (a gift from Yasunori Machida), which was derived from pGreen0029 vector (Hellens et al., 2000), generating pHT035 (pGreen-pPIN1::GUS-GFP) and pHT047 (pGreen-pPHB::GUS-GFP). *BEN2* coding region including a spacer (AEAAAKEAAAKA) was PCR amplified with primers #11459 (5′ -AGAGGCGCGCCAACAATGGTTTTGGTTACGTCTGT-3′) and #11460 (5′-TCACCATGGCCTTAGCAGCAGCCTCCTTAGCAGCAGCCTCA-GCCACCATATGGCTACCTGA-3′), digested with AscI and NcoI, and cloned into AscI and NcoI sites of cloning vectors pHT035 and pHT047.

To generate pGreen-pPIN2::BEN2-GFP and pGreen-pSCR::BEN2-GFP constructs, proPIN2 and proSCR fragments were PCR amplified with primers: #9810 (AAGCGGCCGCATCATTACCAGTACCGAATG), #9811 (TTGTCGACTTTGATTTACTTTTTCCGGCGA), #10968 (AAAGGGCCCCATGGACATTGGAATCGCCA), and #10969 (AAAGTCGACGGAGATTGAAGGGTTGTTGGT), digested with NotI and SalI, and cloned into Bsp120I and SalI sites of the pGreen-pPHB::BEN2-GFP, replacing the *PHB* promoter sequence. To generate transgenic plants, these constructs were transformed into *ben2* and *ben1;ben2* by agrobacterium-mediated floral-dip procedure (Clough and Bent, 1998). Transgenic plants were selected on solid media containing 25 mg/L kanamycin.

### Drug Treatment

BFA (Molecular Probes, B7450) and L-Kynurenine (Kyn, Tokyo Chemical Industry, K0016) was diluted with liquid Arabidopsis medium from 50 mM stock solution in DMSO. For cell wall staining, seedlings were immersed in 4% (w/v) paraformaldehyde in PBS buffer, supplemented with SR2200 (1:500; Renaissance Chemicals), incubated under vacuum for 1h, rinsed with PBS, and mounted with ClearSee solution (Kurihara et al., 2015). For live cell imaging, seedlings were mounted in 1/2 MS medium.

### Immunolocalization and Microscopy

Immunolocalization was performed as described (Kitakura et al., 2017). Briefly, seedlings were fixed with 4% paraformaldehyde in PBS (1h), adhered on MAS-coat slides (Matsunami glass, S9441), permeabilized by sequential treatment with 2.5% to 3.0% driselase (30 min at 37°C) and a mixture of 10% DMSO and 1% NP-40 substitute (1 h). 3% BSA in PBS was used for blocking and dilution of antibodies as follows: goat anti-PIN1 (1:400; Santa Cruz, sc-27163), rabbit andi-PIN2 (1:2000) (Abas et al., 2006), Cy3-conjugated secondary anti-rabbit (1:600; Sigma, C2306) and DyLight 649-conjugated secondary anti-goat (1:400; Jackson Immuno Research, 705-495-147) antibodies. After incubation with the antibody solutions, samples were washed with PBS and mounted in ClearSee solution (Kurihara et al., 2015). Confocal laser-scanning microscopy was performed with a Carl Zeiss LSM710 microscope.

### Root Phenotypic Analysis

For analysis of meristem size, seedlings were fixed and stained with SR2200 (1:500), diluted in a fixative solution (4% paraformaldehyde, 0.05% Triton X100 in PBS buffer). Samples were then mounted with ClearSee solution. At least three biological replicates were performed and similar results were obtained. Quantitative evaluation was performed with ImageJ. Meristem size was defined as distance between QC and cortex cell which expanded rapidly. To evaluate the distribution of CycB1;1-GUS positive cells in the root tips, distance along longitudinal axis from QC to each cell with strong GUS positive signal was measured by using ImageJ. At least 30 cells from 3 to 10 seedlings from each genotype were scored in one experiment. Two independent experiments gave essentially the same results. Statistical analyses were performed by using Excel 2016 (Microsoft) or PRISM 5 (GraphPad) software. We designated significant difference as 0.01 < p-value ≤ 0.05 (*) and p-value ≤ 0.01 (**).

### GUS Staining

For detection of GUS activity, seedlings were rinsed in a pre-incubation buffer [0.1M sodium phosphate buffer (pH 7.0); 2 mM potassium ferrocyanide; 2 mM potassium ferricyanide]. The buffer was then substituted with a GUS-staining buffer supplemented with 0.25 mg/mL 5-Bromo-4-chloro-3-indolyl β-d-glucuronide (x-gluc.) (Rose Scientific, ES-1007-001) and incubated at 37°C in the darkness. After coloration, GUS staining buffer was substituted by 70% ethanol. Seedlings were hydrated and finally soaked in ClearSee solution.

## Results

### Activity of the Root Meristem Is Reduced in *ben1; ben2*

**Figure 1:**
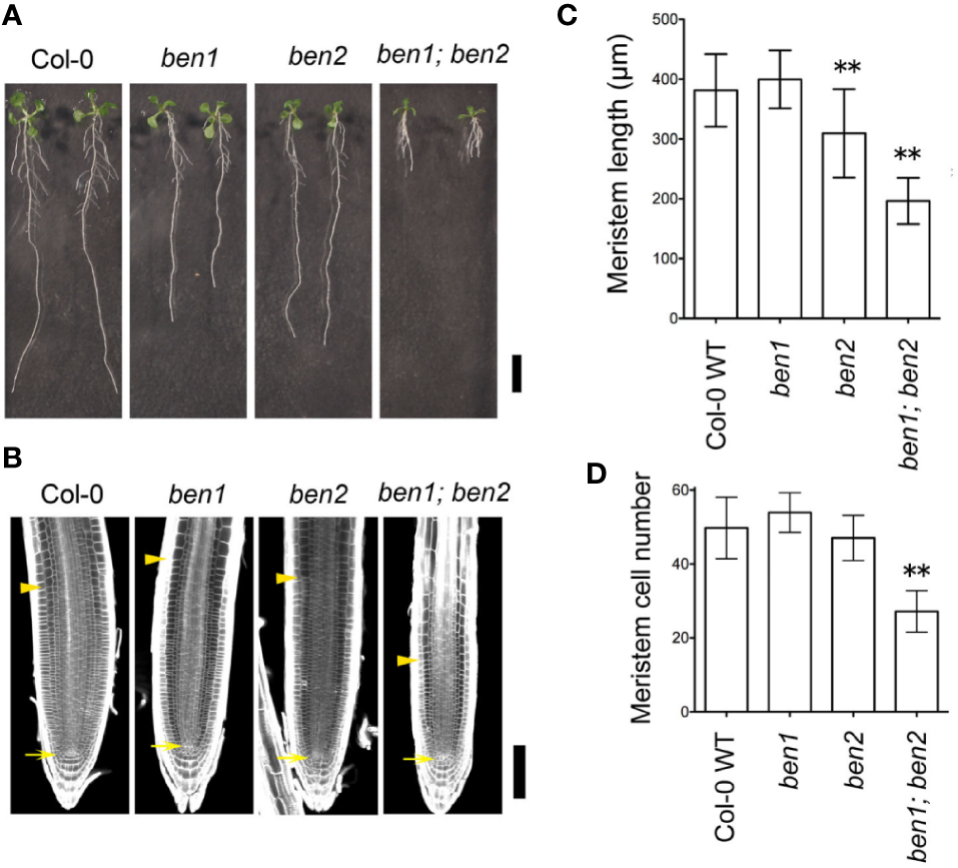
Phenotype of *ben1; ben2* mutant roots. **(A)** Gross morphology of wildtype Col-0, *ben1*, *ben2*, and *ben1; ben2* mutants at 10 days after germination (DAG). **(B–D)** Meristem size of primary roots in Col-0, *ben1*, *ben2*, and *ben1; ben2* at 5 DAG. The arrows and arrowheads in **(B)** indicate the positions of the QC and the boundary between the meristematic zone and the elongation zone of root, respectively. Representative data from at least three independent biological replications are shown. Asterisks (**) in the graphs **(C**, **D)** represent significant difference (p-value ≤ 0.01 by Student's t-test). Error bars represent SD (n ≥ 15 for **(C)** and n ≥ 19 for **(D)**). Scale bars: 10 mm in **(A)**; 100 µm in **(B)**.

Reduced growth of the primary root is an obvious phenotype of the *ben1; ben2* double mutant seedlings (**Figure 1A**). To investigate the developmental basis of the root growth inhibition, we analyzed the meristematic activity of wildtype, *ben1*, *ben2*, and *ben1; ben2* seedling roots. In the wild-type root, relatively small cells with high cell division capacity represent the zone of cell division, or the root meristem, which locates above the quiescent center (QC). *ben1* and *ben2* single mutants showed similar size of meristem and number of cells in meristematic regions compared with wildtype, although *ben2* single mutant had a tendency to have slightly short root meristem. On the other hand, in the *ben1; ben2* double mutants, the size of root meristem and the number of cells in the meristematic regions were reduced as compared with wildtype root (**Figures 1B–D**). These results suggest that cells ongoing cell proliferation at the root tip were decreased in *ben1; ben2*, which could at least in part account for the root growth inhibition in the mutants.

### *ben1; ben2* Double Mutant Is Less Sensitive to Genetic Manipulation of PIN Expression and Pharmacological Inhibition of Auxin Biosynthesis

**Figure 2:**
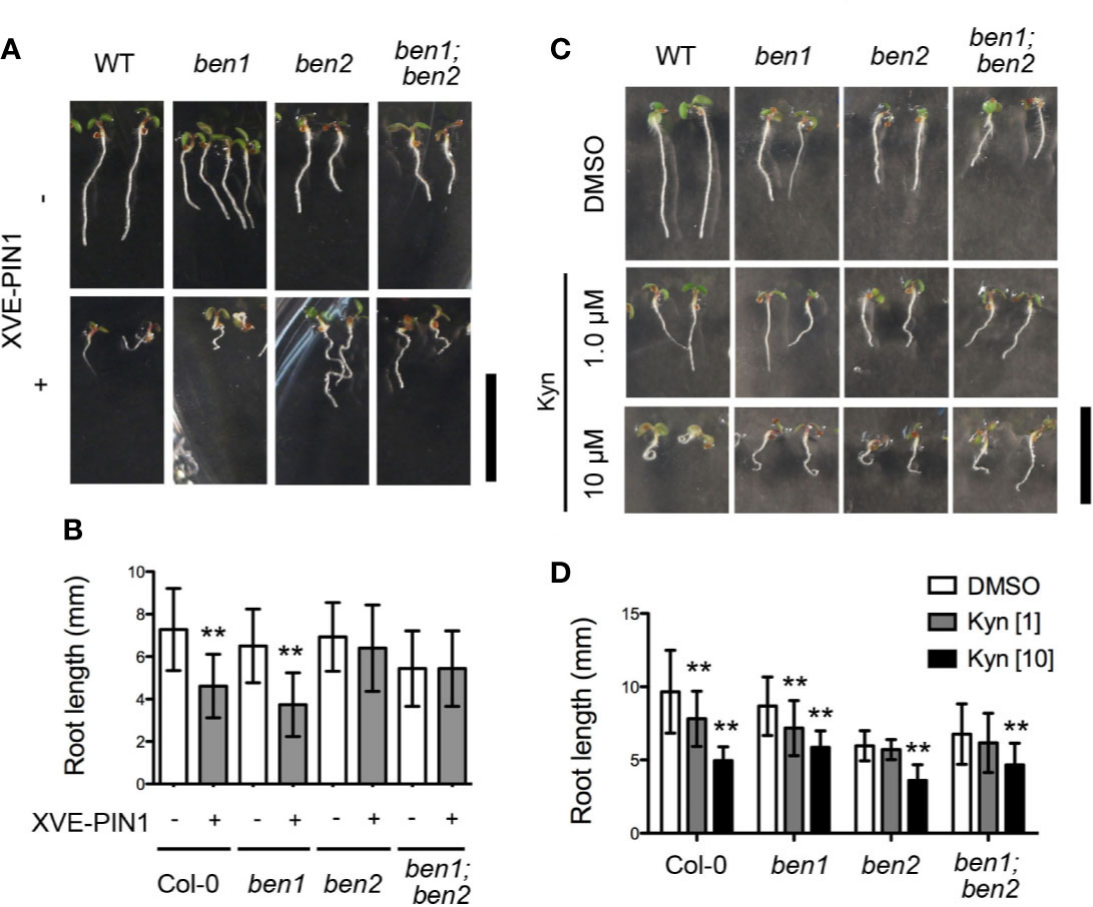
Inhibition of root elongation by compromised auxin transport and biosynthesis. **(A, B)** Morphology of seedlings **(A)** and root length **(B)** of Col-0, *ben1*, *ben2*, and *ben1; ben2* with or without XVE-PIN1 grown in the presence of estradiol (4 µM) at 5 DAG. **(C, D)** Gross morphology of seedlings **(C)** and root length **(D)** of Col-0, *ben1*, *ben2*, and *ben1; ben2* at 5 DAG with treatment of DMSO and Kyn (1.0 µM, and 10 µM). Error bars in **(B**, **D)** represent SD [n ≥ 51 for **(B)**; n ≥ 13 for **(D)**]. Representative results from at least two biological replicates were shown. Asterisks indicate significant difference as compared with corresponding control experiments [0.01 < p-value ≤ 0.05 (*) and p-value ≤ 0.01 (**)]. Scale bars: 10 mm.

In a preceding study, it was indicated that BEN1 and BEN2 function in membrane trafficking of PIN protein (Tanaka et al., 2009). However, it has remained elusive whether the developmental defects observed in *ben1; ben2* double mutants are caused by the abnormality of PIN-dependent auxin distribution. To gain insight into the causal relationship between the growth defects of the *ben1; ben2* double mutants and functionality of PIN proteins and/or auxin distribution, we next tested the genetic relationship between the *ben1; ben2* double mutant and PIN1 overexpression, which is known to affect root growth and meristem size (Mravec et al., 2008). To overexpress PIN1 in the mutant background, an estradiol-inducible PIN1 (XVE-PIN1) was introduced into the *ben1*, *ben2*, and *ben1; ben2* mutants. As reported previously, in wildtype background, the root length was dramatically reduced by overexpressing PIN1 (**Figures 2A, B**). Similarly, *ben1* single mutant showed reduction of root length. On the other hand, in *ben2* and *ben1; ben2* seedlings, growth of the primary root was only moderately inhibited, suggesting that root growth of the double mutant was less sensitive to the overexpression of PIN1 (**Figures 2A, B**).

We further investigated the effect of an auxin biosynthesis inhibitor kynurenine (kyn) on root growth of wildtype, *ben1*, *ben2*, and the *ben1; ben2* mutant seedlings. In wildtype, kyn clearly reduced the root length at 10 µM by 50% on average as compared with DMSO control (**Figures 2C, D**). In contrast, root length of the *ben1*, *ben2*, and *ben1; ben2* mutants was reduced by 30%, 20%, and 30% at 10 µM kyn respectively, indicating that the root growth of the mutant was only mildly inhibited by kyn (**Figures 2C, D**). Based on these observations, we reasoned that the defects of root development in *ben1; ben2* is, at least to some extent, caused by the change of PIN- and auxin-dependent regulation of root meristem and BEN2 might play a particularly important role in regulating PIN-dependent root growth.

### BEN2/VPS45 Is Expressed Throughout Root and Functions Cell Autonomously in PIN Trafficking

**Figure 3:**
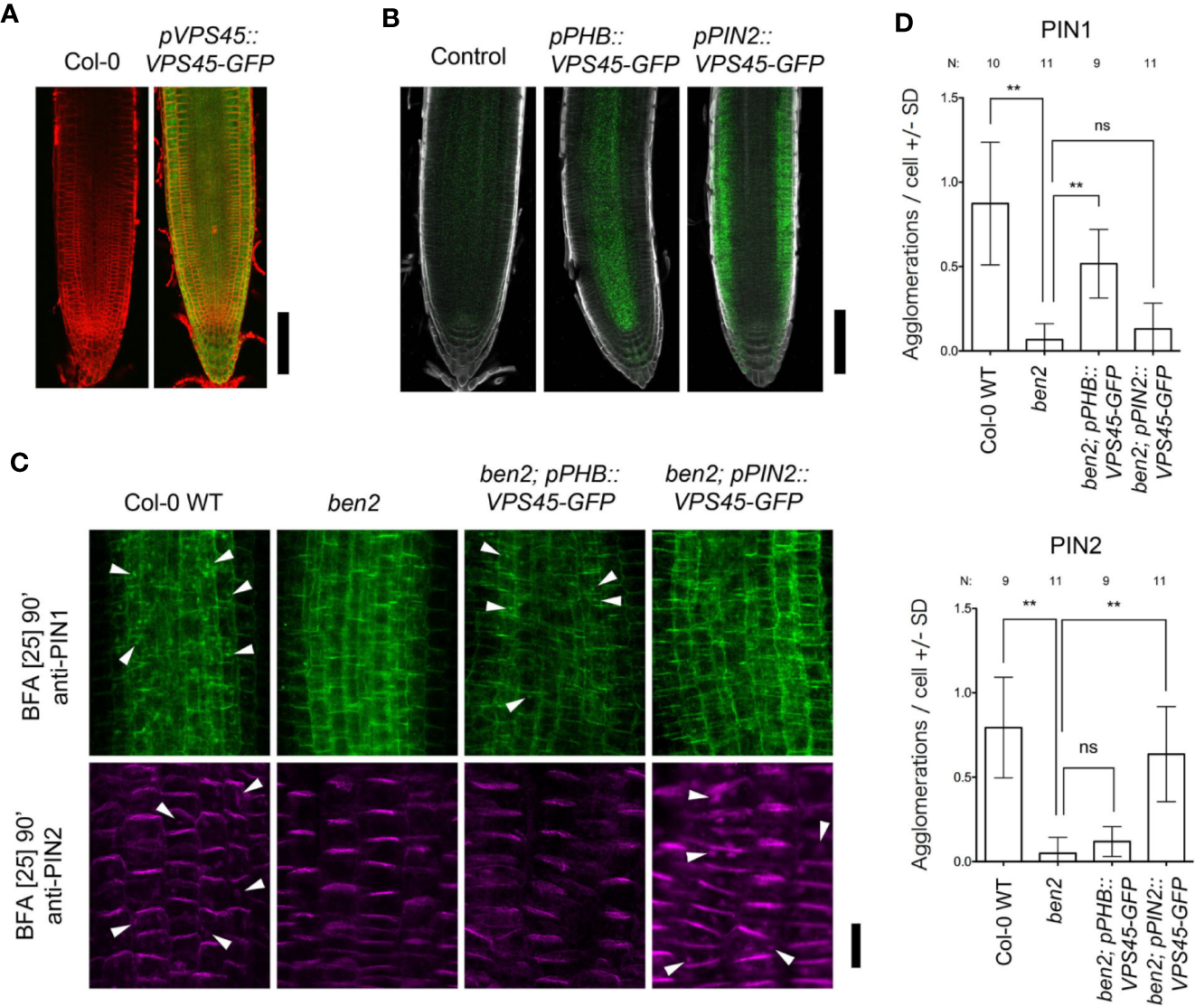
BEN2/VPS45 regulates PIN trafficking cell-autonomously. **(A)** Root tips of Col-0 and *pVPS45::VPS45-GFP* at 7 DAG. **(B)** Root tips of *ben2* single mutant (control) and *ben2* mutants harboring *pPHB::VPS45-GFP* and *pPIN2::VPS45-GFP* constructs at 7 DAG. **(C)** Immunolocalization of PIN1 and PIN2 in BFA-treated Col-0, *ben2*, *ben2*; *pPHB::VPS45-GFP*, and *ben2*; *pPIN2::VPS45-GFP* seedlings. PIN1 localization in vasculature (green signals in upper panels) and PIN2 in epidermis (magenta signals in lower panels) are indicated. Arrowheads indicate agglomeration of PIN proteins. Representative images from at least three independent experiments are shown. **(D)** Quantification of PIN1 and PIN2 agglomeration in the BFA-treated seedling roots. Number of agglomeration (>1 µm2 in cross section) per cell was scored in at least 10 cells in each root. Data from 9 to 11 seedling root (187–378 cells) from each genotype were evaluated. Asterisks (**) represent significant difference (p-value ≤ 0.01 by Student's t-test). Scale bars: 100 µm in **(A, B)** and 20 µm in **(C)**.

We focused on BEN2 function to form root architecture as *ben2* single mutant showed different response against PIN1 overexpression although *ben1* single mutant showed almost same response as seen in wildtype. BEN2 is expressed widely in root tip (**Figure 3A**). To test if BEN2 functions cell-autonomously in regulating PIN trafficking, we established transgenic lines which expressed BEN2 tissue-specifically in *ben2* single mutant background. For this purpose, BEN2 was expressed as GFP fusion protein under the control of either the *PHB* or *PIN2* promoters, which drives tissue-specific gene expression in internal tissues and outer tissues, respectively. Investigation of the GFP signals of the *pPHB::BEN2-GFP* and *pPIN2::BEN2-GFP* transgenic plants revealed tissue-specific expression, consistent with other reports (Vieten et al., 2005; Miyashima et al., 2011; Sebastian et al., 2015) (**Figure 3B**). By using these transgenic lines, *ben2* mutant phenotypes in terms of PIN trafficking was evaluated by using brefeldin A (BFA). It has been shown that, BFA inhibits exocytosis and induces accumulation of PIN1 and PIN2 proteins in the BFA compartments in wildtype cells, whereas the agglomeration of both PIN1 and PIN2 is less pronounced in *ben2* mutant cells (Tanaka et al., 2009; Tanaka et al., 2013). In transgenic lines, BFA compartments visualized with PIN1- and PIN2-antibody were pronounced only in the tissues where BEN2 proteins were supposed to express (**Figures 3C, D**). Together, these results imply that intercellular movement of BEN2-GFP fusion proteins is negligible, and indicate that BEN2-GFP rescued the intracellular PIN trafficking defects of the *ben2* mutant in cell-autonomous manner.

### Expression of BEN2 in Internal Tissues Is Crucial for Root Architecture

**Figure 4:**
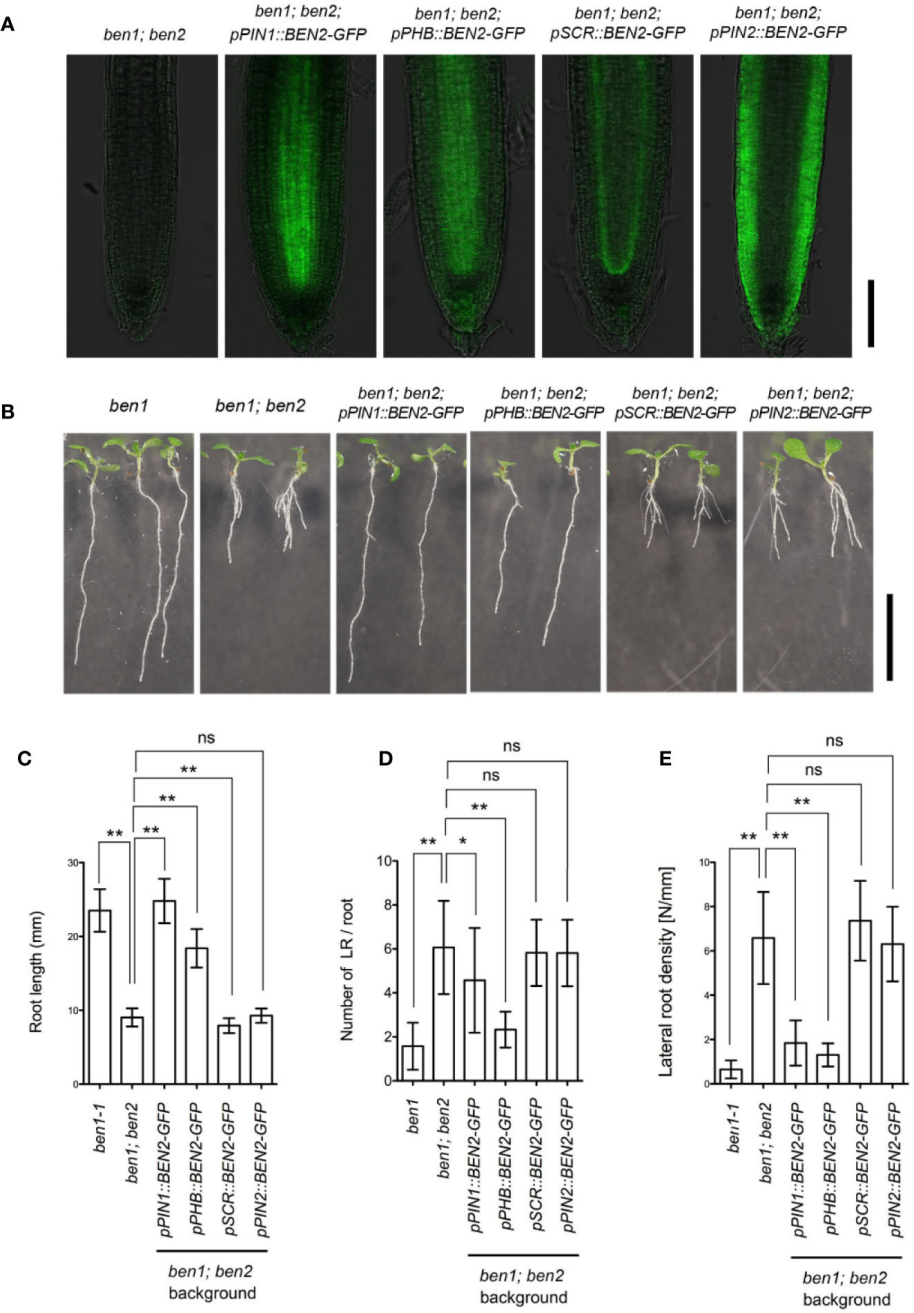
Phenotypes of transgenic plants expressing *BEN2/VPS45-GFP* under control of tissue-specific promoters. **(A)** Expression of BEN2/VPS45-GFP on the *ben1; ben2* double mutant background. Whereas BEN2/VPS45-GFP expressed under *PIN1* and *PHB* promoters were mainly detected in stele, GFP was detected mainly in QC and endodermis in *pSCR::BEN2/VPS45-GFP* and in the outer tissues in *pPIN2::BEN2/VPS45-GFP* lines. Note that *ben1; ben2* double mutant which does not harbor GFP has faint autofluorescence. **(B)** Seedlings of mutants and transgenic lines at 7 DAG. (C-E) Root length **(C)**, number of lateral root per root **(D)**, and lateral root density **(E)** of mutants and transgenic lines at 7 DAG. At least three independent experiments resulted in similar results. Error bars indicate SD (n ≥ 14). Results of statistic evaluation by Mann-Whitney U-test was shown as follows: 0.01 < p-value ≤ 0.05 (*); p-value ≤ 0.01 (**) and not significant (ns). Scale bar: 100 µm in **(A)** and 10 mm in **(B)**.

We next investigated the tissue-specific function of BEN2 on the root architecture. Because the *ben1* and *ben2* single mutant seedlings did not exhibit strong developmental defects, we chose *ben1; ben2* double mutants which exhibit clear morphological defects as the genetic background to express BEN2-GFP under control of tissue-specific promoters. For this experiment, we selected promoters to drive expression in internal tissues (*pPIN1* and *pPHB*), QC, cortex/endodermis initial (CEI) and endodermis (*pSCR*), and outer tissues (*pPIN2*) (**Figure 4A**). As judged by the overall morphology of the root system in young seedlings, *ben1; ben2; pPIN1::BEN2-GFP* and *ben1; ben2; pPHB::BEN2-GFP*, the root architecture was similar to *ben1* single mutant, indicating that expression of BEN2-GFP in the internal tissues significantly rescued the root growth defect (**Figures 4B–E**). On the contrary, in *ben1; ben2; pSCR::BEN2-GFP* and *ben1; ben2; pPIN2::BEN2-GFP*, the growth defect of the primary roots did not recover (**Figures 4B–E**). These results indicated that BEN2 expressed in inner tissues has crucial role for root architecture.

### Cell Proliferation and Auxin Distribution in the Tip of Roots Depend on BEN2 Expressed in Inner Tissue

**Figure 5:**
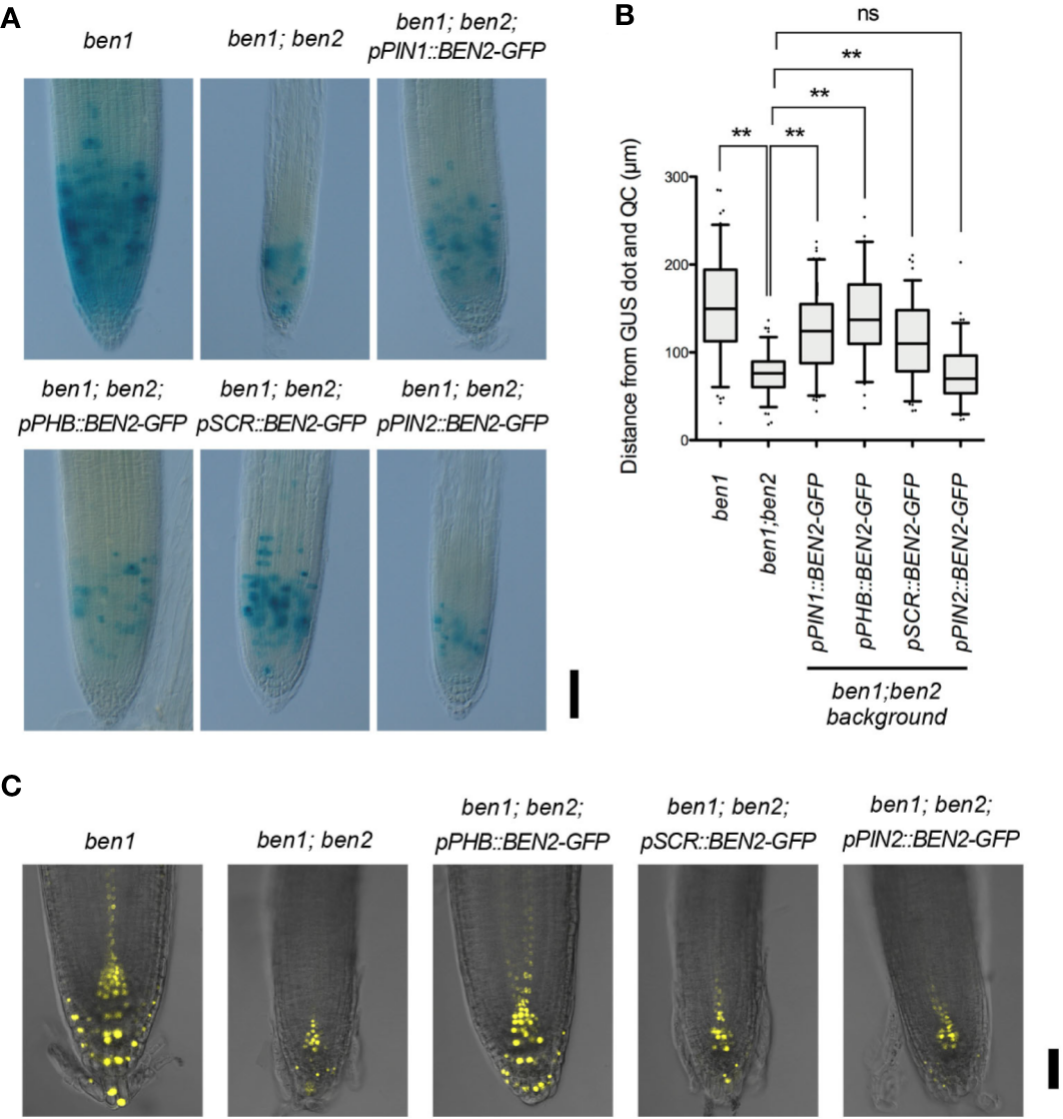
Expression patterns of *CycB1;1-GUS* and *DR5rev::Venus-N7* in transgenic lines expressing *BEN2/VPS45-GFP* driven by tissue-specific promoters. **(A)** Root tips of mutants and the transgenic lines expressing *CycB1;1-GUS* at 7 DAG. Three independent experiments resulted in similar results. **(B)** Distribution patterns of cells with strong CycB1;1-GUS activity in mutants and transgenic lines expressing BEN2/VPS45-GFP, shown as distance from QC at 7 DAG. Location of each GUS-positive cell was measured and presented as boxes (25–75 percentile) and whiskers (5–95 percentile). The dots represent outliers. The graph represents the data from the independent experiments (n ≥ 73). Results of statistic evaluation was shown as follows: 0.01 < p-value ≤ 0.05 (*); p-value ≤ 0.01 (**) and not significant (ns). **(C)** Auxin response distribution in root tips of *ben1* and *ben1; ben2* mutants, and *ben1; ben2* double mutants harboring *BEN2/VPS45-GFP* constructs, as visualized by *DR5rev::Venus-N7* at 7 DAG. Scale bars: 100 µm in **(A)** and 50 µm in **(C)**.

To investigate the recovery mechanism of *ben1; ben2* by BEN2 expressed tissue-specifically, we introduced CycB1;1-GUS marker, which indicates the transition from G2 to M phase, into the mutants and transgenic lines. Under our condition, strong GUS signals were mainly detected in the cells within approximately 100 to 200 µm above the QC in the *ben1* mutant background. In the *ben1; ben2* roots, however, the cells expressing GUS was confined in a smaller region, which was typically within less than 100 µm above the QC (**Figure 5A**). These results suggest that *ben1; ben2; CycB1;1-GUS* shows reduction of cell proliferation compared with *ben1; CycB1;1-GUS*. The region expressing CycB1;1-GUS in root tip of *ben1; ben2; pPIN1::BEN2-GFP; CycB1;1-GUS* and *ben1; ben2; pPHB::BEN2-GFP; CycB1;1-GUS* was similar to that of *ben1; CycB1;1-GUS* (**Figures 5A, B**). On the other hand, *ben1; ben2; pPIN2::BEN2-GFP; CycB1;1-GUS* exhibited CycB1;1-GUS expression in confined regions close to the QC, patterns of which were indistinguishable from those observed in *ben1; ben2; CycB1;1-GUS* (**Figures 5A, B**). The pattern of GUS-positive cells of *ben1; ben2; pSCR::BEN2-GFP; CycB1;1-GUS* was intermediate of those observed in the *ben1* single mutant and *ben1; ben2* double mutant background, indicating that *pSCR::BEN2-GFP* moderately recovered the cell division activity in the root tip (**Figures 5A, B**).

To examine how the tissue-specific recovery of BEN2 affected the auxin response maxima, we introduced an auxin-response reporter *DR5rev::3xVenus-N7* in the mutants and transgenic lines. Characterization of Venus expression revealed that, while strong *DR5* activity was detected in the root tip of *ben1* single mutant background in typical patterns, expression of *DR5rev::3xVenusN7* was apparently reduced in the *ben1; ben2* double mutant background. Together with the results of previous observation of the patterns of *DR5rev::GFP* in the mutants (Tanaka et al., 2013), we reasoned that *ben2* mutation, in concert with *ben1* mutation, has affected auxin distribution and hence *DR5* expression. The patterns of *DR5rev::3xVenusN7* expression in the *ben1; ben2; pSCR::BEN2-GFP* and *ben1; ben2; pPIN2::BEN2-GFP* background were similar to those in *ben1; ben2* double mutant background, which showed narrow expression pattern at the tips of roots. In contrast, *DR5* activity in the *ben1; ben2; pPHB::BEN2-GFP* background was similar to that in the *ben1* single mutant (**Figure 5C**). These results suggested that expression of BEN2 in the internal tissues including stele is relevant in auxin distribution at the root tip and in sustaining cell proliferation.

## Discussion

In this paper, we investigated the developmental roles of membrane trafficking components BEN1/BIG5 ARF GEF and BEN2/VPS45 Sec1/Munc18 protein. It has been indicated that BEN1 and BEN2 are involved in membrane trafficking of PIN protein and *ben1;ben2* double mutants exhibit defects in auxin-response gradient and various developmental processes including growth of primary root (Tanaka et al., 2013). To know whether the developmental defects of *ben1; ben2* is caused by the disruption of PIN polarity and auxin distribution, we investigated the relationship by overexpressing PIN1 which is known to have effect on root growth (Mravec et al., 2008). As reported, the root elongation in Col-0 was inhibited when expression of PIN1 was induced (**Figure 2**). In contrast, induction of PIN1 expression had only moderate effects on inhibition of root growth in the *ben1; ben2* background. We also checked the relationship between root elongation and auxin biosynthesis with an auxin biosynthesis inhibitor kyn. While kyn strongly inhibited the root growth of Col-0, root growth of *ben1; ben2* was only moderately inhibited (**Figure 2**). These results indicated that the abnormality of *ben1; ben2* root development might have been caused by the defects of PIN expression or localization, and auxin distribution. In this scenario, defects in PIN localization and reduced auxin accumulation are relevant for the root growth defect, and these might not be further inhibited by PIN1 overexpression and the auxin biosynthesis inhibitor. Thus, our results corroborate the developmental roles of the membrane traffic components BEN1 and BEN2 in regulation of root architecture through PIN polarity and auxin distribution. Curiously, growth of *ben2* single mutant but not that of *ben1* was insensitive to XVE-PIN1, which could be attributed to the different trafficking steps affected by these two mutations (Tanaka et al., 2013) or to the presence of redundant components (Singh et al., 2018).

The clear developmental defects in *ben1;ben2* double mutant as well as PIN1-insensitive *ben2* mutant phenotypes prompted us to further investigate the developmental role of BEN2 by expressing BEN2 under control of various tissue-specific promoters. Our results showed that BEN2 regulates PIN trafficking cell-autonomously (**Figure 3**) and that the tissue-specific expression of BEN2 had differential effects on recovery of *ben1; ben2* root development defects (**Figures 4** and **5**), allowing us to evaluate the developmental role of BEN2 in specific tissues. Phenotypic studies indicated that root length and lateral root density were consistently recovered when BEN2-GFP was expressed in internal tissues either by PIN1 or PHB promoters. Based on these results, we concluded that BEN2 expressed in inner tissues mainly regulates root elongation and lateral root formation. In previous studies, it has been suggested that growth of seedling root involves regulators of PIN expression or localization in internal tissues, such as embryonic provascular tissue and vascular tissue of seedling root (Dello Ioio et al., 2008; Wolters et al., 2011). In other report, it was suggested that the density of lateral root is determined by auxin pulse from lateral root cap (Xuan et al., 2016). In our study, BEN2 expression in the inner tissues was relevant for regulation of root growth and lateral root formation. Thus, our results are in good agreement with the crucial roles of internal tissues in supporting PIN-dependent regulation of root growth. Concerning the dense lateral root phenotype of the *ben1; ben2* double mutants, it would be possible that the spread of PIN1 polarity to lateral side of the PM induced auxin leakage to outer tissues, and this might have interfered with auxin pulse.

Our findings bring next question how BEN2 in inner tissues regulates root development. To investigate it, we checked the activity of auxin distribution and cell proliferation of transgenic plants. To visualize them, we introduced *DR5rev::Venus-N7* and *CycB1;1-GUS* into the transgenic lines. It has been reported that regulation of PIN expression and cell cycle is of central importance in regulating root meristem size in Arabidopsis. As such, alterations of auxin distribution and root meristem sizes often correlate with changes in root growth (Blilou et al., 2005; Dello Ioio et al., 2008; Takahashi et al., 2013). Auxin distribution, as judged by *DR5rev::Venus-N7*, looked to be recovered with BEN2 in inner tissues but not in other tissues (**Figure 5C**). Transgenic lines which expressed BEN2 in inner tissues showed recovery of cell proliferation zone, too. On the other hand, BEN2 expressed in outer tissues did not contribute to the recovery of meristematic activity (**Figure 5B**). In this respect, our analysis revealed correlation between enlarged zone of cell division and recovery of root growth. To our surprise, however, *pSCR::BEN2-GFP*, which drove BEN2 expression in endodermis and QC, seemed to moderately expanded the zone of cell division without significantly stimulating root growth (**Figure 5**). As root length is determined not only by the cell number but also cell length (Rahman et al., 2007; Yang et al., 2017; Ashraf and Rahman, 2019), it is possible that root elongation is also affected in the transgenic lines expressing BEN2 tissue-specifically. Interestingly, exogenously applied auxin or altered growth conditions significantly change the balance between cell proliferation, cell elongation, and root growth (Rahman et al., 2007; Yang et al., 2017). Thus it would be interesting to test whether the tissue-specific BEN2 expression affects any context-dependent developmental mechanism for root growth.

In summary, our results suggest that BEN2 has important roles in supporting root growth through PIN-dependent auxin distribution. Using tissue-specific promoters, we showed that BEN2 expressed in inner tissues sufficiently contributes to maintain the auxin distribution and cell proliferation in the root tip. Thus the role of BEN2 in root development can be explained mainly by regulation of tip-ward auxin transport through inner tissues, which affects root elongation and lateral root formation.

## Data Availability Statement

All datasets presented in this study are included in the article.

## Author Contributions

HT, YM, and TK developed the concept of the research. YM, HT, and MS prepared the research materials. YM, KO, NF, and HT performed experiments. All authors contributed to the article and approved the submitted version.

## Funding

This work has been supported in part by the Japanese Society for the Promotion of Science (JSPS) KAKENHI [16H04806 to HT; 20H04886 to TK]; program for Leading Graduate School for Osaka University: Interdisciplinary Program for Biomedical Sciences (IPBS) [to YM].

## Conflict of Interest

The authors declare that the research was conducted in the absence of any commercial or financial relationships that could be construed as a potential conflict of interest.

## References

[B1] Abas L. Benjamins R. Malenica N. Paciorek T. Wiśniewska J. Moulinier-Anzola J. C.. (2006). Intracellular trafficking and proteolysis of the Arabidopsis auxin-efflux facilitator PIN2 are involved in root gravitropism. Nat. Cell Biol. 8, 249–256. doi: 10.1038/ncb1369, PMID: 16489343

[B2] Ashraf M. A. Rahman A. (2019). Cold stress response in Arabidopsis thaliana is mediated by GNOM ARF-GEF. Plant J. 97, 500–516. doi: 10.1111/tpj.14137, PMID: 30362633

[B3] Banda J. Bellande K. von Wangenheim D. Goh T. Guyomarc'h S. Laplaze L.. (2019). Lateral Root Formation in Arabidopsis: A Well-Ordered LRexit. Trends Plant Sci. 24, 826–839. doi: 10.1016/j.tplants.2019.06.015, PMID: 31362861

[B4] Blilou I. Xu J. Wildwater M. Willemsen V. Paponov I. Frimi J.. (2005). The PIN auxin efflux facilitator network controls growth and patterning in Arabidopsis roots. Nature 433, 39–44. doi: 10.1038/nature03184, PMID: 15635403

[B5] Casanova-Sáez R. Voß U. (2019). Auxin Metabolism Controls Developmental Decisions in Land Plants. Trends Plant Sci. 24, 741–754. doi: 10.1016/j.tplants.2019.05.006, PMID: 31230894

[B6] Clough S. J. Bent A. F. (1998). Floral dip: A simplified method for Agrobacterium-mediated transformation of Arabidopsis thaliana. Plant J. 16, 735–743. doi: 10.1046/j.1365-313x.1998.00343.x, PMID: 10069079

[B7] Colón-Carmona A. You R. Haimovitch-Gal T. Doerner P. (1999). Spatio-temporal analysis of mitotic activity with a labile cyclin-GUS fusion protein. Plant J. 20, 503–508. doi: 10.1046/j.1365-313x.1999.00620.x, PMID: 10607302

[B8] Dello Ioio R. Nakamura K. Moubayidin L. Perilli S. Taniguchi M. Morita M. T.. (2008). A genetic framework for the control of cell division and differentiation in the root meristem. Science 322, 1380–1384. doi: 10.1126/science.1164147, PMID: 19039136

[B9] Friml J. Vieten A. Sauer M. Weijers D. Schwarz H. Hamann T.. (2003). Efflux-dependent auxin gradients establish the apical-basal axis of Arabidopsis. Nature 426, 147–153. doi: 10.1038/nature02085, PMID: 14614497

[B10] Geldner N. Anders N. Wolters H. Keicher J. Kornberger W. Muller P.. (2003). The Arabidopsis GNOM ARF-GEF mediates endosomal recycling, auxin transport, and auxin-dependent plant growth. Cell 112, 219–230. doi: 10.1016/S0092-8674(03)00003-5, PMID: 12553910

[B11] Grunewald W. Friml J. (2010). The march of the PINs: developmental plasticity by dynamic polar targeting in plant cells. EMBO J. 29, 2700–2714. doi: 10.1038/emboj.2010.181, PMID: 20717140 PMC2924653

[B12] Heisler M. G. Ohno C. Das P. Sieber P. Reddy G. V. Long J. A.. (2005). Patterns of auxin transport and gene expression during primordium development revealed by live imaging of the Arabidopsis inflorescence meristem. Curr. Biol. 15, 1899–1911. doi: 10.1016/j.cub.2005.09.052, PMID: 16271866

[B13] Hellens R. P. Anne Edwards E. Leyland N. R. Bean S. Mullineaux P. M. (2000). pGreen: A versatile and flexible binary Ti vector for Agrobacterium-mediated plant transformation. Plant Mol. Biol. 42, 819–832. doi: 10.1023/A:1006496308160, PMID: 10890530

[B14] Huang F. Zago M. K. Abas L. Marion A. Van, Galván-ampudia C. S. van Marion A.. (2010). Phosphorylation of conserved PIN motifs directs Arabidopsis PIN1 polarity and auxin transport. Plant Cell 22, 1129–1142. doi: 10.1105/tpc.109.072678, PMID: 20407025 PMC2879764

[B15] Kitakura S. Adamowski M. Matsuura Y. Santuari L. Kouno H. Arima K.. (2017). BEN3/BIG2 ARF GEF is involved in brefeldin a-sensitive trafficking at the trans-golgi network/early endosome in arabidopsis thaliana. Plant Cell Physiol. 58, 1801–1811. doi: 10.1093/pcp/pcx118, PMID: 29016942

[B16] Kleine-Vehn J. Dhonukshe P. Sauer M. Brewer P. B. Wiśniewska J. Paciorek T.. (2008). ARF GEF-Dependent Transcytosis and Polar Delivery of PIN Auxin Carriers in Arabidopsis. Curr. Biol. 18, 526–531. doi: 10.1016/j.cub.2008.03.021, PMID: 18394892

[B17] Kurihara D. Mizuta Y. Sato Y. Higashiyama T. (2015). ClearSee: a rapid optical clearing reagent for whole-plant fluorescence imaging. Development 142, 4168–4179. doi: 10.1242/dev.127613, PMID: 26493404 PMC4712841

[B18] Ljung K. Hull A. K. Celenza J. Yamada M. Estelle M. Normanly J.. (2005). Sites and regulation of auxin biosynthesis in arabidopsis roots. Plant Cell 17, 1090–1104. doi: 10.1105/tpc.104.029272, PMID: 15772288 PMC1087988

[B19] Miyashima S. Koi S. Hashimoto T. Nakajima K. (2011). Non-cell-autonomous microRNA 165 acts in a dose-dependent manner to regulate multiple differentiation status in the Arabidopsis root. Development 138, 2303–2313. doi: 10.1242/dev.060491, PMID: 21558378

[B20] Mockaitis K. Estelle M. (2008). Auxin receptors and plant development: a new signaling paradigm. Annu. Rev. Cell Dev. Biol. 24, 55–80. doi: 10.1146/annurev.cellbio.23.090506.123214, PMID: 18631113

[B21] Mravec J. Kubeš M. Bielach A. Gaykova V. Petrášek J. Skůpa P.. (2008). Interaction of PIN and PGP transport mechanisms in auxin distribution-dependent development. Development 135, 3345–3354. doi: 10.1242/dev.021071, PMID: 18787070

[B22] Naramoto S. Otegui M. S. Kutsuna N. de Rycke R. Dainobu T. Karampelias M.. (2014). Insights into the localization and function of the membrane trafficking regulator GNOM ARF-GEF at the Golgi apparatus in Arabidopsis. Plant Cell 26, 3062–3076. doi: 10.1105/tpc.114.125880, PMID: 25012191 PMC4145132

[B23] Petricka J. J. Winter C. M. Benfey P. N. (2012). Control of Arabidopsis Root Development. Annu. Rev. Plant Biol. 63, 563–590. doi: 10.1146/annurev-arplant-042811-105501, PMID: 22404466 PMC3646660

[B24] Rahman A. Bannigan A. Sulaman W. Pechter P. Blancaflor E. B. Baskin T. (2007). Auxin, actin and growth of the Arabidopsis thaliana primary root. Plant J. 50, 514–528. doi: 10.1111/j.1365-313X.2007.03068.x, PMID: 17419848

[B25] Sauer M. Kleine-Vehn J. (2019). PIN-FORMED and PIN-LIKES auxin transport facilitators. Development 146, dev168088. doi: 10.1242/dev.168088, PMID: 31371525

[B26] Sebastian J. Ryu K. H. Zhou J. Tarkowská D. Tarkowski P. Cho Y. H.. (2015). PHABULOSA Controls the Quiescent Center-Independent Root Meristem Activities in Arabidopsis thaliana. PloS Genet. 11, 1–27. doi: 10.1371/journal.pgen.1004973, PMID: 25730098 PMC4346583

[B27] Singh M. K. Richter S. Beckmann H. Kientz M. Stierhof Y. D. Anders N.. (2018). A single class of ARF GTPase activated by several pathway-specific ARF-GEFs regulates essential membrane traffic in Arabidopsis. PloS Genet. 14, e1007795. doi: 10.1371/journal.pgen.1007795, PMID: 30439956 PMC6264874

[B28] Sugawara S. Mashiguchi K. Tanaka K. Hishiyama S. Sakai T. Hanada K.. (2015). Distinct Characteristics of Indole-3-Acetic Acid and Phenylacetic Acid, Two Common Auxins in Plants. Plant Cell Physiol. 56, 1641–1654. doi: 10.1093/pcp/pcv088, PMID: 26076971 PMC4523386

[B29] Takahashi N. Kajihara T. Okamura C. Kim Y. Katagiri Y. Okushima Y.. (2013). Cytokinins control endocycle onset by promoting the expression of an APC/C activator in arabidopsis roots. Curr. Biol. 23, 1812–1817. doi: 10.1016/j.cub.2013.07.051, PMID: 24035544

[B30] Tanaka H. Dhonukshe P. Brewer P. B. Friml J. (2006). Spatiotemporal asymmetric auxin distribution: A means to coordinate plant development. Cell Mol. Life Sci. 63, 2738–2754. doi: 10.1007/s00018-006-6116-5, PMID: 17013565 PMC11136431

[B31] Tanaka H. Kitakura S. De Rycke R. De Groodt R. Friml J. (2009). Fluorescence Imaging-Based Screen Identifies ARF GEF Component of Early Endosomal Trafficking. Curr. Biol. 19, 391–397. doi: 10.1016/j.cub.2009.01.057, PMID: 19230664

[B32] Tanaka H. Kitakura S. Rakusová H. Uemura T. Feraru M. I. de Rycke R.. (2013). Cell Polarity and Patterning by PIN Trafficking through Early Endosomal Compartments in Arabidopsis thaliana. PloS Genet. 9, e1003540. doi: 10.1371/journal.pgen.1003540, PMID: 23737757 PMC3667747

[B33] Tanaka H. Nodzynski T. Kitakura S. Feraru M. I. Sasabe M. Ishikawa T.. (2014). BEX1/ARF1A1C is required for BFA-sensitive recycling of PIN auxin transporters and auxin-mediated development in arabidopsis. Plant Cell Physiol. 55, 737–749. doi: 10.1093/pcp/pct196, PMID: 24369434 PMC3982122

[B34] Vanneste S. Friml J. (2009). Auxin: A Trigger for Change in Plant Development. Cell 136, 1005–1016. doi: 10.1016/j.cell.2009.03.001, PMID: 19303845

[B35] Vieten A. Vanneste S. Wiśniewska J. Benková E. Benjamins R. Beeckman T.. (2005). Functional redundancy of PIN proteins is accompanied by auxin-dependent cross-regulation of PIN expression. Development 132, 4521–4531. doi: 10.1242/dev.02027, PMID: 16192309

[B36] Wiśniewska J. Xu J. Seifertová D. Brewer P. B. Ruůžička K. Blilou I.. (2006). Polar PIN Localization Directs Auxin Flow in Plants. Science 312, 883. doi: 10.1126/science.1121356, PMID: 16601151

[B37] Wolters H. Anders N. Geldner N. Gavidia R. Jürgens G. (2011). Coordination of apical and basal embryo development revealed by tissue-specific GNOM functions. Development 138, 117–126. doi: 10.1242/dev.059147, PMID: 21138974

[B38] Xuan W. Band L. R. Kumpf R. P. Van Damme D. Parizot B. De Rop G.. (2016). Cyclic programmed cell death stimulates hormone signaling and root development in Arabidopsis. Science 351, 384–387. doi: 10.1126/science.aad2776, PMID: 26798015

[B39] Yang X. Dong G. Palaniappan K. Mi G. Baskin T. I. (2017). Temperature-compensated cell production rate and elongation zone length in the root of Arabidopsis thaliana. Plant Cell Environ. 40, 264–276. doi: 10.1111/pce.12855, PMID: 27813107

[B40] Zhang X. Adamowski M. Marhava P. Tan S. Zhang Y. Rodriguez L.. (2020). Arabidopsis Flippases Cooperate with ARF GTPase Exchange Factors to Regulate the Trafficking and Polarity of PIN Auxin Transporters. Plant Cell 32, 1644–1664. doi: 10.1105/tpc.19.00869, PMID: 32193204 PMC7203944

[B41] Zwiewka M. Bilanovičová V. Seifu Y. W. Nodzyński T. (2019). The Nuts and Bolts of PIN Auxin Efflux Carriers. Front. Plant Sci. 10, 985. doi: 10.3389/fpls.2019.00985, PMID: 31417597 PMC6685051

